# Persistence and evolution of *Pseudomonas aeruginosa* following initiation of highly effective modulator therapy in cystic fibrosis

**DOI:** 10.1128/mbio.00519-24

**Published:** 2024-04-02

**Authors:** Catherine R. Armbruster, Yasmin K. Hilliam, Anna C. Zemke, Samar Atteih, Christopher W. Marshall, John Moore, Junu Koirala, Leah Krainz, Jordan R. Gaston, Stella E. Lee, Vaughn S. Cooper, Jennifer M. Bomberger

**Affiliations:** 1 Department of Microbiology and Molecular Genetics, University of Pittsburgh School of Medicine, Pittsburgh, Pennsylvania, USA; 2 Department of Microbiology and Immunology, Geisel School of Medicine, Dartmouth College, Hanover, New Hampshire, USA; 3 Division of Pulmonary, Allergy and Critical Care Medicine, University of Pittsburgh Medical Center, Pittsburgh, Pennsylvania, USA; 4 Department of Pediatrics, University of Pittsburgh School of Medicine, Pittsburgh, Pennsylvania, USA; 5 Department of Biological Sciences, Marquette University, Milwaukee, Wisconsin, USA; 6 Department of Otolaryngology, University of Pittsburgh Medical Center, Pittsburgh, Pennsylvania, USA; Baylor College of Medicine, Houston, Texas, USA

**Keywords:** cystic fibrosis, *Pseudomonas aeruginosa*, evolution, pathogenesis, respiratory pathogens, lung infection

## Abstract

Today, more than 90% of people with cystic fibrosis (pwCF) are eligible for the highly effective cystic fibrosis transmembrane conductance regulator (CFTR) modulator therapy called elexacaftor/tezacaftor/ivacaftor (ETI) and its use is widespread. Given the drastic respiratory symptom improvement experienced by many post-ETI, clinical studies are already underway to reduce the number of respiratory therapies, including antibiotic regimens, that pwCF historically relied on to combat lung disease progression. Early studies suggest that bacterial burden in the lungs is reduced post-ETI, yet it is unknown how chronic *Pseudomonas aeruginosa* populations are impacted by ETI. We found that pwCF remain infected throughout their upper and lower respiratory tract with their same strain of *P. aeruginosa* post-ETI, and these strains continue to evolve in response to the newly CFTR-corrected airway. Our work underscores the continued importance of CF airway microbiology in the new era of highly effective CFTR modulator therapy.

**IMPORTANCE:**

The highly effective cystic fibrosis transmembrane conductance regulator modulator therapy Elexakaftor/Tezacaftor/Ivacaftor (ETI) has changed cystic fibrosis (CF) disease for many people with cystic fibrosis. While respiratory symptoms are improved by ETI, we found that people with CF remain infected with *Pseudomonas aeruginosa*. How these persistent and evolving bacterial populations will impact the clinical manifestations of CF in the coming years remains to be seen, but the role and potentially changing face of infection in CF should not be discounted in the era of highly effective modulator therapy.

## OBSERVATION

Highly effective cystic fibrosis transmembrane conductance regulator (CFTR) modulator therapy (HEMT), including elexacaftor/tezacaftor/ivacaftor (ETI), is now approved for more than 90% of adults with the genetic disorder cystic fibrosis. ETI dramatically reduces upper and lower respiratory symptoms and improves outcomes, yet it is unclear whether established bacterial infections in the airways of people with CF (pwCF) on ETI are eradicated (1–4). One challenge of this new era of HEMT is that many pwCF fail to produce sputum that is needed to monitor airway infections by opportunistic pathogens such as *Pseudomonas aeruginosa*. A prior study of the CFTR modulator ivacaftor also showed improvement of respiratory symptoms, but *P. aeruginosa* was not eradicated (5). Sputum *P. aeruginosa* density and total bacterial burden decreased in adults following initiation of ivacaftor, but *P. aeruginosa* rebounded after 1 year of treatment and individuals remained infected with their same strain of *P. aeruginosa* as determined by multilocus sequence typing (MLST) and pulsed-field gel electrophoresis (PFGE). Here, we used the latest culture-independent genomic methods to determine whether *P. aeruginosa* persists in the respiratory tract of pwCF following initiation of ETI and how *P. aeruginosa* evolves to persist in the new CFTR-corrected niche.

**Fig 1:**
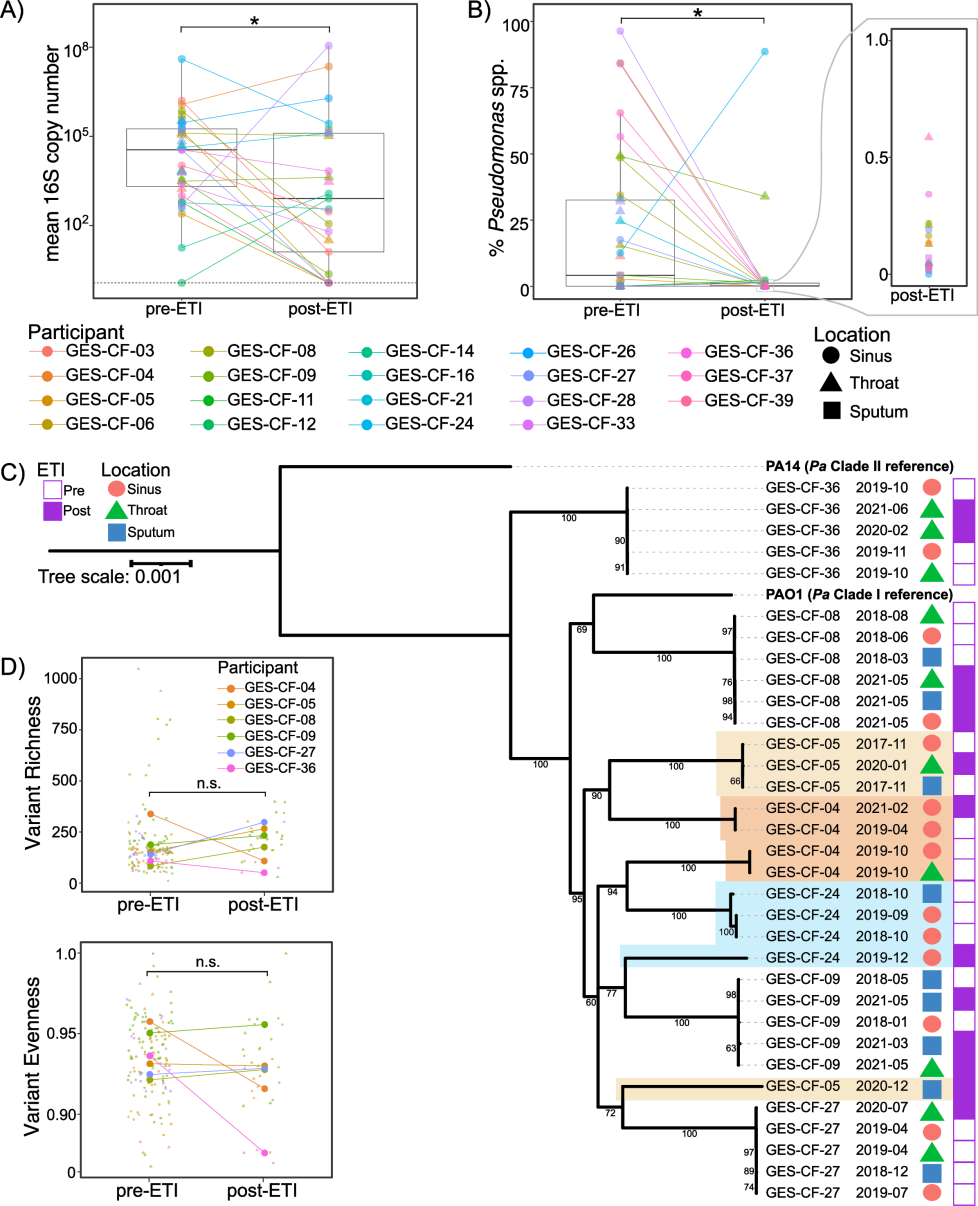
Clonal populations of *P. aeruginosa* persist throughout the respiratory tract of adults with CF following initiation of highly effective modulator therapy. (A) Total bacterial load is lower in most participants’ post-ETI samples compared to pre-ETI. *P < 0.05 (mixed effects linear regression). (B) Relative abundance of *Pseudomonas* spp. is reduced post-ETI, but remains detectable in all but one study participant. *P < 0.05 (mixed effects linear regression). (C) The same *P. aeruginosa* clones present pre-ETI persist post-ETI. Core genome phylogeny of metagenome-assembled *P. aeruginosa* genomes from seven individuals. The reference strains PAO1 and PA14 are included as representatives of Clade I and Clade II *P. aeruginosa* strains, respectively. Colored shading indicates instances where a single study participant’s P. *aeruginosa* does not form a monophyletic clade. Dates are the year and month the sample was collected. (D) Richness and evenness of genetic variants in *P. aeruginosa* populations is unchanged post-ETI. Top = single nucleotide polymorphism (SNP) richness. Bottom = Pieleou’s evenness. n.s. = not significant (*P* > 0.05 by mixed effects linear regression).

We performed 16S rRNA gene amplicon sequencing of sinus, throat, and/or sputum samples from 19 adults with CF collected before and after initiation of ETI (Table 1) under protocol CR19100149-006 approved by the Institutional Review Board at the University of Pittsburgh. Sinus swabs were collected endoscopically and sputum was spontaneously expectorated. In 16 out of 19 individuals, only sinus and/or throat swabs were available post-ETI because these individuals were no longer able to spontaneously expectorate sputum. While total bacterial load was lower in individuals’ post-ETI samples (Fig. 1A), *Pseudomonas* spp. remained detectable by 16S amplicon sequencing in 18 out of 19 individuals (Fig. 1B). We confirmed that this amplicon sequence variant represented *P. aeruginosa* by performing amplicon sequencing on the species-specific genes encoding Pel, Psl, and/or ExoS (Table S1). See Supplemental text for additional methodological details.

**TABLE 1:** Characteristics of the cohort

Parameter	16S cohort (*n* = 19)		Pre/post-ETI *P. aeruginosa* metagenome subset (*n* = 7 of 19)	Non-ETI *P. aeruginosa* metagenome cohort (*n* = 7)
Median age on enrollment, years (range)	33.8 (21.9–48)		32.2 (26.2–48)	26.4 (21.2–49.7)
Male, no. (%)	8/19 (42.1)		3/7 (42.9)	4/7 (57.1)
CFTR genotype, no. (%)				
ΔF508 homozygous	12/19 (63.2)		5/7 (71.4)	1/7 (14.3)
ΔF508/other	7/19 (36.8)		2/7 (28.6)	5/7 (71.4)
Unknown	--		--	1/7 (14.3)
BMI on enrollment (range)	22.3 (17–35.9)		22.5 (17.1–35.9)	21.1 (18.7–34.9)
ppFEV1 on enrollment (range)	56 (21–103)		43 (21–71)	71 (30–110)
FESS prior to enrollment (range)^*a*^	14/19 (73.7)		5/7 (71.4)	7/7 (100)
Transplant prior to enrollment (range)	1/19 (5.3)		0/7 (0)	1/7 (14.3)
No. (%) using modulator upon enrollment				
Ivacaftor	1/19 (5.3)		1/7 (14.3)	1/7 (14.3)
Orkambi	1/19 (5.3)		1/7 (14.3)	0/7 (0)
Symdeko	2/19 (10.5)		1/7 (14.3)	0/7 (0)
Days from enrollment to ETI prescription, median (range)	608 (1–875)		671 (48–840)	--
Days from ETI prescription to post-ETI sample, median (range)	372 (15–566)		342 (15–566)	--
Days from enrollment to last *P. aeruginosa* metagenome, median (range)	--		1,099 (348–1,245)	624 (266–798)

^*a*^ FESS = functional endoscopic sinus surgery.

After observing that *P. aeruginosa* persists in the respiratory tract post-ETI in virtually the entire cohort, we next sought to determine whether pwCF remain infected post-ETI by the same clonal lineage(s) of *P. aeruginosa* that they harbored pre-ETI or whether they acquired new strains from the environment. *P. aeruginosa* was cultured from respiratory samples pre- and post-ETI in seven individuals and subjected to whole population genomic sequencing. Phylogenomic analyses revealed that six of the seven individuals remained infected by their same pre-ETI *P. aeruginosa* genotype (Fig. 1C; Table S2). In one individual (GES-CF-04), *P. aeruginosa* populations fell into two separate clades prior to ETI, but *P. aeruginosa* from only one of these clades was detected post-ETI. We cannot rule out that this strain remained present post-ETI at levels below what was detectable by our population sequencing approach. However, the apparent loss of this strain could either be due to changing environmental conditions in the airways post-ETI favoring one strain over the other or random drift leading to loss of one strain during the population bottleneck. We detected evidence of newly infecting strains post-ETI in two participants. In GES-CF-24, their pre- and post-ETI samples did not fall into the same clade, indicating a strain replacement occurred coincident with initiation of ETI. In GES-CF-05, a new *P. aeruginosa* strain was detected post-ETI, along with the pre-ETI strain. Taken together, we observed that most patients retained their original lineage of *P. aeruginosa* after initiating ETI therapy. Given this, we sought to determine whether and how changes to the mucosal environment post-ETI alter the course of evolution in these persistent *P. aeruginosa* populations.

**Fig 2:**
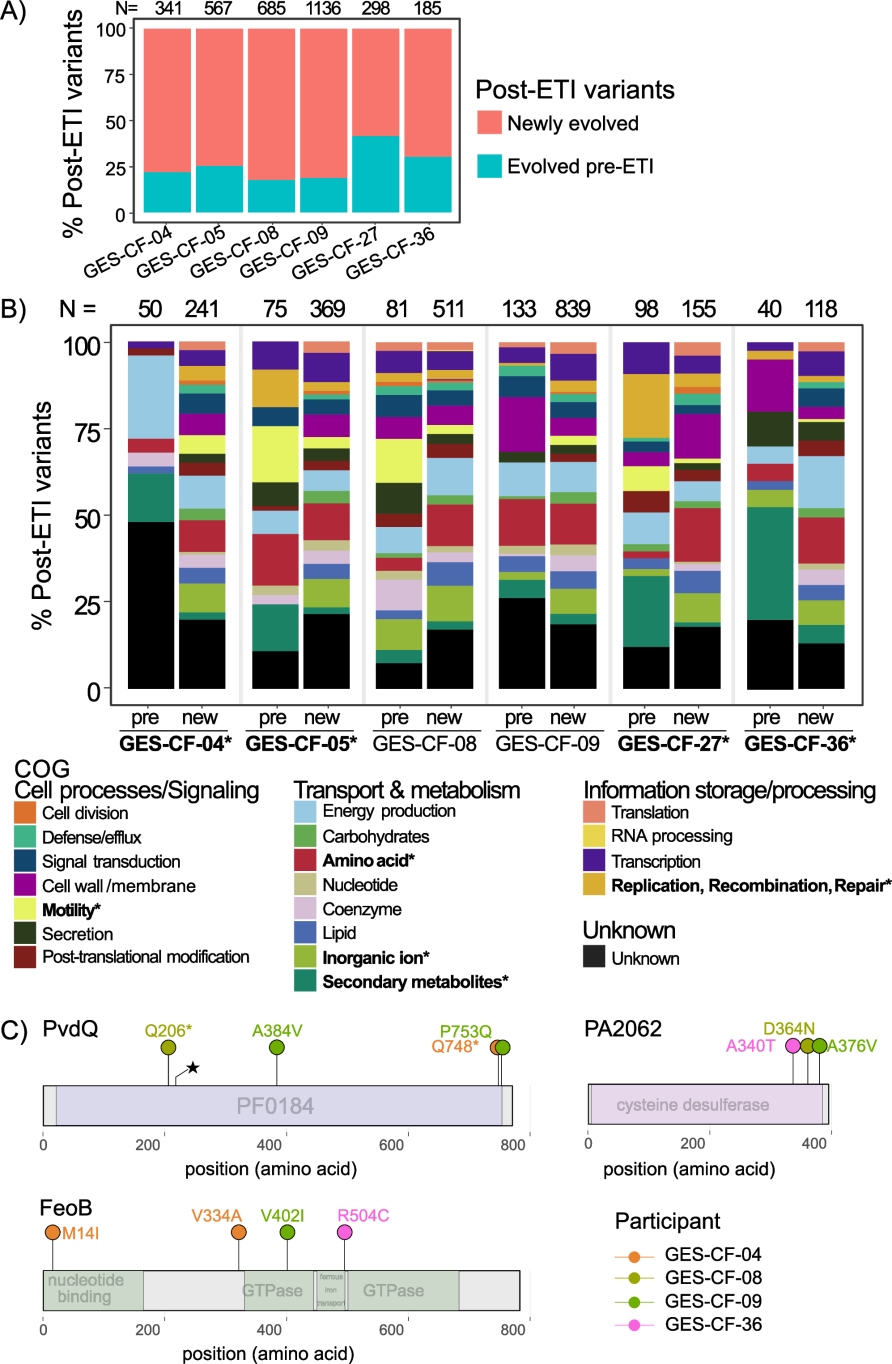
**(A**) Most post-ETI variants were not previously detected pre-ETI. Red = proportion of post-ETI variants that were never detected pre-ETI in each study participant. Teal = proportion of post-ETI variants that were also present in pre-ETI populations. The numbers above each bar are the counts of unique post-ETI variants, including those in intergenic regions. (**B**) Functional categories of newly evolved variants differ from variants that had evolved pre-ETI. Bars show the proportion of post-ETI variants that are assigned to each functional category (COG) by eggNOG. For each participant, left bar (pre) =post-ETI variants that were previously detected pre-ETI. Right bar = post-ETI variants that were newly evolved post-ETI (new). The numbers above each bar are the counts of unique post-ETI variants within coding regions of genes that were either present pre-ETI or newly evolved post-ETI. Bolded labels with asterisks on the COG category labels indicate categories that significantly differed within at least one study participant (Fisher’s exact test, *P* < 0.05). Bolded labels with asterisks on participant labels indicate individuals whose COG categories were significantly different (Fisher’s exact test, *P* < 0.05). (**C**) Lollipop diagram of genes that were mutated post-ETI, but not pre-ETI, in three or more study participants. Colors of variants indicate the study participant in which each mutation was detected.

To measure the impact of ETI use on *P. aeruginosa* evolution, we first asked how the bottleneck imposed by ETI therapy impacted genetic diversity of persistent *P. aeruginosa* populations. We expected that the observed population bottleneck would be associated with a loss of genetic diversity either due to bottleneck-induced drift or a selective sweep of one or very few clones from pre-ETI that survived the sudden change in the airway environment post-ETI. Therefore, we looked for a reduction in counts of single nucleotide polymorphisms (SNPs) and/or changes to the distribution of variants post-ETI. We were surprised to find no significant difference in SNP richness (counts of variants) or evenness (distribution of variants in the populations; Fig. 1D). One interpretation is that despite a reduction in population size, ETI did not alter the airway environment significantly enough to alter selection on existing variants. Alternatively, although richness and evenness were the same post-ETI, the mutations themselves could be different if the post-ETI environment provides new selective pressures. We examined whether each post-ETI variant had evolved pre-ETI or whether it was newly evolved post-ETI. Remarkably, the *P. aeruginosa* populations found in all study participants continued to evolve new variants post-ETI, suggesting novel adaptations to the new selective pressures in the CFTR-corrected airway environment (Fig. 2A). In a control cohort of pwCF who did not take ETI and were colonized by *P. aeruginosa*, fewer new mutations were detected over a similar time span. This finding suggests that ETI generates novel selective pressures beyond expectations of continued evolution by *P. aeruginosa* in pwCF (Fig. S1).

We also found that the functional categories of genes newly mutated post-ETI differed from those of the mutations that had evolved pre-ETI in four of the six participants (Fisher’s exact tests, *P* < 0.05; Fig. 2B). In all four of these individuals, the proportion of novel mutations occurring post-ETI in genes involved in secondary metabolite biosynthesis was significantly changed. This category includes genes required for the production of siderophores that are used to acquire iron from the environment. Specifically, genes required for the production of the siderophores pyoverdine and/or pyochelin were newly mutated post-ETI in three people. This pathway-level trend across multiple study participants suggests that selection on iron acquisition strategies has changed in the post-ETI respiratory environment. To determine more specifically whether ETI imposes a common set of new selective pressures, we examined whether any genes were undergoing parallel evolution across multiple study participants post-ETI. Parallel evolution, mutations that independently arise across multiple study participants, provides strong evidence of selection. We identified three genes that were exclusively mutated post-ETI in three or more individuals: *pvdQ*, *feoB*, and PA2062 (Table S3; Fig. 2C; permutation tests, *P* < 0.05). Two of these genes are involved in iron acquisition from the environment (*pvdQ* and *feoB*). PA2062 shares homology with the cysteine desulfurase, *IscS*, and is implicated in thiamine metabolism. Parallel evolution of these genes exclusively post-ETI suggests changes in nutrient availability or competition for these nutrients (e.g., when microbial population sizes are smaller) in the post-ETI airway environment.

In conclusion, we found that the total bacterial load and relative abundance of *P. aeruginosa* are reduced following the initiation of ETI. However, *P. aeruginosa* remains detectable by amplicon sequencing throughout the respiratory tract in most individuals. Furthermore, our whole population genomic analyses revealed that the same strain of *P. aeruginosa* present prior to ETI persists post-ETI, raising the possibility that the sinuses could serve as an alternative site for monitoring respiratory microbes in the absence of expectorated sputum. We found that *P. aeruginosa* undergoes a population bottleneck coincident with the initiation of ETI, followed by drastic changes to the mutational spectra of the *P. aeruginosa* population due to new selective pressures in the post-ETI airway environment. This bottleneck is likely explained by changes in the CF respiratory environment caused by corrected CFTR function, which we have recently reported (6); anecdotally, some individuals with advanced CF lung disease experienced severe cough and copious sputum production in the first few days post-ETI (7). The widespread use and success of HEMT in CF has sparked an interest in changes to CF disease management toward reducing the medication burden in people taking ETI (8). Our study reveals that pwCF on ETI remain infected with *P. aeruginosa* that continues to evolve post-ETI, and that new *P. aeruginosa* strains can also infect post-ETI. How these persistent and evolving bacterial populations will impact the clinical manifestations of CF in the coming years remains to seen, but the role and potentially changing face of infection in CF should not be discounted in the era of HEMT. More work is needed to determine how these populations continue to change post-ETI, including whether population sizes increase after the initial bottleneck, and how best to balance the treatment burden in the face of respiratory symptom improvement with the ongoing need for infection monitoring and control.

## Data Availability

Raw reads from the 16S amplicon and *P. aeruginosa* population sequences are available in NCBI’s SRA under BioProject PRJNA1081394.

## SUPPLEMENTAL MATERIAL

Figure S1 mbio.00519-24-s0001.pdf In a time-matched control cohort of individuals who had not taken ETI, a greater percentage of variants from later time points were present at earlier time points as compared to Fig. 2A.

Supplemental text mbio.00519-24-s0002.docx Supplemental materials and methods.

Table S1 mbio.00519-24-s0003.xlsx Results of amplicon sequencing.

Table S2 mbio.00519-24-s0004.xlsx MLST types of consensus genomes.

Table S3 mbio.00519-24-s0005.xlsx Parallel evolved genes among six study participants.

ASM does not own the copyrights to Supplemental Material that may be linked to, or accessed through, an article. The authors have granted ASM a non-exclusive, world-wide license to publish the Supplemental Material files. Please contact the corresponding author directly for reuse.

## References

[1] Nichols DP, Paynter AC, Heltshe SL, Donaldson SH, Frederick CA, Freedman SD, Gelfond D, Hoffman LR, Kelly A, Narkewicz MR, Pittman JE, Ratjen F, Rosenfeld M, Sagel SD, Schwarzenberg SJ, Singh PK, Solomon GM, Stalvey MS, Clancy JP, Kirby S, Van Dalfsen JM, Kloster MH, Rowe SM, PROMISE Study group. 2022. Clinical effectiveness of elexacaftor/tezacaftor/ivacaftor in people with cystic fibrosis: a clinical trial. Am J Respir Crit Care Med 205:529–539. doi:10.1164/rccm.202108-1986OC 34784492 PMC8906485

[2] Schnell A, Hober H, Kaiser N, Ruppel R, Geppert A, Tremel C, Sobel J, Plattner E, Woelfle J, Hoerning A. 2023. Elexacaftor – Tezacaftor – Ivacaftor treatment improves systemic infection parameters and Pseudomonas aeruginosa colonization rate in patients with cystic fibrosis a monocentric observational study. Heliyon 9:e15756. doi:10.1016/j.heliyon.2023.e15756 37153441 PMC10160512

[3] Sheikh S, Britt RD, Ryan-Wenger NA, Khan AQ, Lewis BW, Gushue C, Ozuna H, Jaganathan D, McCoy K, Kopp BT. 2023. Impact of elexacaftor–tezacaftor–ivacaftor on bacterial colonization and inflammatory responses in cystic fibrosis. Pediatr Pulmonol 58:825–833. doi:10.1002/ppul.26261 36444736 PMC9957929

[4] Long DR, Holmes EA, Goss CH, Singh PK, Waalkes A, Salipante SJ. 2023. Cell-free DNA detects P. aeruginosa lung infection in modulator-treated people with cystic fibrosis. Am J Respir Crit Care Med 208:944–947. doi:10.1164/rccm.202305-0844LE 37540570 PMC10870864

[5] Hisert KB, Heltshe SL, Pope C, Jorth P, Wu X, Edwards RM, Radey M, Accurso FJ, Wolter DJ, Cooke G, Adam RJ, Carter S, Grogan B, Launspach JL, Donnelly SC, Gallagher CG, Bruce JE, Stoltz DA, Welsh MJ, Hoffman LR, McKone EF, Singh PK. 2017. Restoring cystic fibrosis transmembrane conductance regulator function reduces airway bacteria and inflammation in people with cystic fibrosis and chronic lung infections. Am J Respir Crit Care Med 195:1617–1628. doi:10.1164/rccm.201609-1954OC 28222269 PMC5476912

[6] Atteih SE, Armbruster CR, Hilliam Y, Rapsinski GJ, Bhusal JK, Krainz LL, Gaston JR, DuPont M, Zemke AC, Alcorn JF, Moore JA, Cooper VS, Lee SE, Forno E, Bomberger JM. 2024. Effects of highly effective modulator therapy on the dynamics of the respiratory mucosal environment and inflammatory response in cystic fibrosis. Pediatr Pulmonol n/a. doi:10.1002/ppul.26898 PMC11058019 38353361

[7] Ramos KJ, Pilewski JM, Taylor-Cousar JL. 2021. Challenges in the use of highly effective modulator treatment for cystic fibrosis. J Cyst Fibros 20:381–387. doi:10.1016/j.jcf.2021.01.007 33531206 PMC8192344

[8] Mayer-Hamblett N, Nichols DP, Odem-Davis K, Riekert KA, Sawicki GS, Donaldson SH, Ratjen F, Konstan MW, Simon N, Rosenbluth DB, Retsch-Bogart G, Clancy JP, VanDalfsen JM, Buckingham R, Gifford AH. 2021. Evaluating the impact of stopping chronic therapies after modulator drug therapy in cystic fibrosis: the SIMPLIFY clinical trial study design. Ann Am Thorac Soc 18:1397–1405. doi:10.1513/AnnalsATS.202010-1336SD 33465316 PMC8513667

