# Supplementary figures and images for: Persistence and evolution of Pseudomonas aeruginosa following initiation of highly effective modulator therapy in cystic fibrosis
Source: mBio. 2024 Apr 2;15(5):e00519-24. doi: 10.1128/mbio.00519-24 (PMC11077959; doi:10.1128/mbio.00519-24)

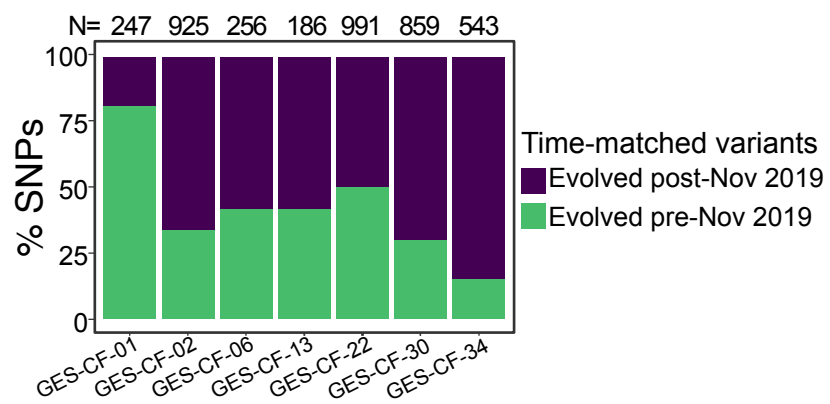

Supplement: Figure S1 — In a time-matched control cohort of individuals who had not taken ETI, a greater percentage of variants from later time points were present at earlier time points as compared to Fig. 2A. [file mbio.00519-24-s0001.pdf]
