# Supplementary material for: Persistence and evolution of Pseudomonas aeruginosa following initiation of highly effective modulator therapy in cystic fibrosis
Source: mBio. 2024 Apr 2;15(5):e00519-24. doi: 10.1128/mbio.00519-24 (PMC11077959; doi:10.1128/mbio.00519-24)
Supplement: Supplemental text — Supplemental materials and methods. [file mbio.00519-24-s0002.docx]

***Study design, participants, and sample collection***

We performed a prospective, longitudinal study of 38 CF adults with symptomatic CRS and prior functional endoscopic sinus surgery (FESS) following an IRB-approved protocol (CR19100149-006) between November 2017 and June 2021 at a CF-focused otolaryngology clinic at the University of Pittsburgh. Inclusion criteria for the present study required the study participant to have at least one sinus, throat, or sputum microbiota sample prior to ETI that was positive for *P. aeruginosa* by 16S amplicon sequencing and at least one sinus, throat, or sputum microbiota sample sequenced post-ETI (n = 19 individuals; **Table 1**). We sampled the sinuses, throat, and sputum of study participants during quarterly clinic visits and unscheduled visits. Two swabs were collected endoscopically from the sinuses: one for rRNA gene amplicon sequencing (dry flock swab; Puritan Medical Products, Guilford, ME) and the other for bacterial culturing (BD CultureSwab MaxV(+); Becton, Dickinson, and Company, Franklin Lakes, NJ). At each subsequent visit, the same location was sampled from each participant’s sinuses to minimize sampling bias. Spontaneously expectorated sputum was collected in sterile, pre-weighed specimen containers (Electron Microscopy Sciences, Hatfield, PA). Throat swabs were collected prior to sputum samples or instead of a sputum sample, if a participant was unable to produce sputum (BD CultureSwab MaxV(+); Becton, Dickinson, and Company, Franklin Lakes, NJ). All specimens were stored at 4^o^C and processed within 4 hours of sampling. Visit dates are truncated to year and month to maintain privacy of participants.

***Microbiota DNA extraction Quantitative Polymerase Chain Reaction (qPCR) to quantify total bacterial load***

DNA was extracted from dry flock swabs (sinus), culture swabs (throat), or sputum as previously described^1^, with the following modifications: hypotonic lysis for sinus and throat swabs was performed by incubation at room temperature for 15 min in 1350 µL distilled water; benzonase treatment of sinus and throat swabs was carried out in 1.5 mL final volume. Environmental and extraction kit controls were extracted alongside experimental samples. Prior to DNA extraction, sputum was weighed, treated with 1mL of Sputasol (Thermo Fisher Scientific Remel Products, Lenexa, KS) per gram of sputum, and incubated shaking at 37^o^C and 200rpm for 15 minutes solubilize. DNA concentration was quantified with a Qubit 4 fluorimeter (Invitrogen, Waltham, MA). Total bacterial load was quantified by quantitative polymerase chain reaction (qPCR) of the V4 region of the 16S rRNA gene as previously described using validated primers 515f (GTGYCAGCMGCCGCGGTAA ) and 806r (GGACTACNVGGGTWTCTAAT ) modified by Walters *et al*.^2^

***16S rRNA gene amplicon sequencing and analyses***

Sequencing of the V4 region of the 16S rRNA gene after amplification with primers 515f and 806r was performed on a NovaSeq 6000 by the DNA Services Lab at University of Illinois at Urbana-Champaign (Illinois, USA). The cycle conditions were: 251 cycles using a NovaSeq 500-cycle sequencing kit v1.5. Paired end 2x250 reads were demultiplexed, quality checked, and trimmed by QIIME2 and DADA2^3,4^. Chimeras were identified and removed by USEARCH.^5^ Rarefaction was performed to a sampling depth of 11500 by QIIME2. Classification was performed using the SILVA 138.1 rRNA database. Environmental and extraction kit controls were sequenced alongside experimental samples.

***Verification of the Pseudomonas spp. taxon as P. aeruginosa by multiplexed amplicon sequencing***

Multiplex amplicon sequencing of the *pelA* (primers: TTCATCAAGCCCTATCC and CCATCTTGTAGCCATACT), *pslA* (primers: GATGTACACGGAAAGGA and CGGAACAGGATGTAGAG), and *exoS* (primers: GAGGTCAGCAGAGTATC and AGTCTTCACTACCTGTTC) genes was performed by the DNA Services Lab at University of Illinois at Urbana-Champaign (Illinois, USA). The cycle conditions were: 251 cycles using a NovaSeq 500-cycle sequencing kit v1.5.

***P. aeruginosa* population culturing and genomic DNA extraction**

Each sample type was swabbed (sinus and throat) or spread (sputum; 100μL of Sputastol-treated sputum) in triplicate onto *Pseudomonas* Isolation Agar (Millipore, Burlington, MA) plates. PIA plates were incubated at 37^o^C for 48 hours. All colonies present on the triplicate PIA plates were scraped and resuspended 10 mL phosphate buffered saline (PBS; if more than 100 colonies on plate) or 2 mL PBS (if less than 100 colonies). These triplicate populations were vortexed for 10 minutes, then a 1 mL aliquot of each resuspended bacterial population was pelleted for DNA extraction with a Qiagen DNeasy Blood and Tissue kit (Qiagen, Hilden, Germany).

***P. aeruginosa population sequencing and metagenome-assembled genome (MAG) assembly***

Genomic DNA extracted from triplicate bacterial populations grown on PIA was sequenced by Microbial Genome Sequencing Center (MiGS; Pittsburgh, PA) to generate at least 650 Mbp of 2 x 151 bp paired-end reads using the Illumina NextSeq 2000 platform. Reads were trimmed with Trimmomatic version 0.36 and quality checked with FastQC version 0.11.5.^6,7^ Reads were taxonomically classified with kraken version 2.1.2.^8^ For samples with greater than 10% *P. aeruginosa* reads, reads were assembled into MAGs with SPAdes version 3.12.0 with the optional flag “--meta”.^9^ For most study visits, duplicate or triplicate sequenced populations were available for each body site (because samples were plated in triplicate on PIA, extracted, and sequenced separately). When duplicate or triplicate populations were sequenced, a separate assembly was generated for each population. Contigs smaller than 10kB were removed. These consensus *P. aeruginosa* assemblies represent the dominant clone of *P. aeruginosa* at each timepoint. Assemblies were annotated using prokka version 1.14.6.^10^ For each open reading frame, the homologous *P. aeruginosa* strain PAO1 locus tag was identified using a nucleotide BLAST.^11^ Ultimately, we sequenced *P. aeruginosa* populations both pre- and post-ETI for seven people (**Table 1**). The non-ETI cohort in **Supplemental figure 1** consists of seven people who either did not take ETI during our study or, in the case of GES-CF-06, for whom post-ETI *P. aeruginosa* populations were unavailable.

***Phylogenetic analyses of dominant P. aeruginosa clones from P. aeruginosa populations***

We used roary version 3.12.0 to create a multiFASTA alignment of core genes in a subset of *P. aeruginosa* consensus assemblies.^12^ From each participant, we chose one sinus, throat, and sputum assembly (when available) from both pre-ETI and post-ETI, prioritizing assemblies constructed from samples with the highest percent *P. aeruginosa* reads (kraken) to target assemblies with the longest contigs and read highest coverage. Prior to roary, *P. aeruginosa* contigs were isolated from any potential contaminating species that also grew on PIA using SprayNPray.^13^ We used RAxML version 8.2.4 to construct the phylogenetic tree in **Figure 1C** from the core_gene_alignment.aln file generated by roary.^14^ Branches with a bootstrap value less than 30 were collapsed. To determine whether participants whose *P. aeruginosa* did not form a monophyletic clade were infected by multiple strains of *P. aeruginosa*, we determined the multilocus sequence type (MLST) of each assembly using FastMLST (**Supplemental Table 2**).^15^

***Variant calling in P. aeruginosa populations***

We extracted reads classified by kraken as Pseudomonadales or Pseudomonadaceae or *Pseudomonas* spp. or *Pseudomonas aeruginosa* using seqtk 1.3-r106 before variant calling. We identified mutations present in each *P. aeruginosa* population relative to each participant’s earliest sinus assembly (cleaned by SprayNPray to remove any non-*P. aeruginosa* contigs) and their frequencies using breseq version 0.35.0 in population mode.^16^ When duplicate or triplicate sinus assemblies were available from the earliest study visit, we chose the assembly from the sample with the highest percent *P. aeruginosa* reads as determined by kraken. We included the optional breseq flag “--maximum-read-mismatches 2” to further limit read mapping from contaminating bacterial species that also grew on PIA. Variants present at 100% frequency when mapping reads from the same population used to construct the reference genome were removed. Variants called in visit dates with a newly infecting *P. aeruginosa* strain post-ETI in GES-CF-05 and GES-CF-24 are excluded from **Figure 2** panels A, B, and C.

***Ecological and functional comparisons of mutations pre vs. post-ETI***

Mutational richness in **Figure 1D** was calculated from all variants detected by breseq using the function estimate() and R package vegan.^17^ Pielou’s evenness was calculated by dividing the Shannon diversity (calculated with diversity()) by the natural log of mutational richness. Variant frequencies were normalized by a factor of 10 to generate integers prior to calculating Pieleou’s evenness. Functional categories of genes with non-synonymous mutations were determined with eggNOG. Non-synonymous mutations that were present post-ETI were binned based on whether they initially evolved pre-ETI or were newly evolved post-ETI. These mutations were assigned to a COG category based on the gene they impacted. The count for each COG category was determined for each of six participants (GES-CF-04, -05, -08, -09, -27, and -36) and used as input for the Fisher’s exact tests. A permutation test was performed using the R packages Biostrings and dplyr to determine the likelihood that mutations in the parallel evolved genes in **Supplemental table 3** were due to random chance.^18,19^ For the non-ETI control cohort in **Supplemental figure 1**, populations were binned by timepoint based on whether they were sampled before or after ETI was FDA-approved in the USA (October 2019), in an effort to generate a time-matched comparison with the ETI cohort. For two individuals, a sample was not available after October 2019, so we took their last sample date for their later bin.

***Study approval***

This study was approved by the University of Pittsburgh’s IRB under protocol CR19100149-006.

***Statistics***

Statistical analyses were performed in R. Reduction in bacterial biomass and relative abundance of the *Pseudomonas* spp. taxon post-ETI was determined in **Figures 1AB** by a mixed effects linear regression model with the R packages lme4 and lmerTest to control for repeated measures within study participants. A mixed effects linear regression model was also used to test for differences in mutational richness and evenness while controlling for repeated measures within participants in **Figure 1D**. To determine whether the counts of mutations in each COG category differed for post-ETI mutations that were present pre-ETI versus those that evolved post-ETI for each person in **Figure 2B**, a Fisher’s exact test was performed with the option: simulate.p.value=TRUE. To identify the specific COG categories that are differentially represented in post-ETI mutations that were either present pre-ETI or post-ETI mutations that were newly evolved, row-wise Fisher’s exact tests were performed with the R package rstatix. The FDR-adjusted p-value was used to identify significantly changed COG categories. The permutation test in **Supplemental table 3** was performed with 10,000 trials to randomly simulate the indicated number of mutations occurring on the *P. aeruginosa* PAO1 genome. The p value was determined empirically from the number of genes in each trial with at least the indicated number of mutations (2 or 3) divided by the number of trials. This represents the probability that mutations in the same gene could be present in multiple participants due to random chance rather than selection.

Supplemental References

1. Nelson, M. T. *et al.* Human and Extracellular DNA Depletion for Metagenomic Analysis of Complex Clinical Infection Samples Yields Optimized Viable Microbiome Profiles. *Cell Rep.* **26**, 2227-2240.e5 (2019).

2. Walters William *et al.* Improved Bacterial 16S rRNA Gene (V4 and V4-5) and Fungal Internal Transcribed Spacer Marker Gene Primers for Microbial Community Surveys. *mSystems* **1**, e00009-15 (2015).

3. Callahan, B. J. *et al.* DADA2: High-resolution sample inference from Illumina amplicon data. *Nat. Methods* **13**, 581–583 (2016).

4. Bolyen, E. *et al.* Reproducible, interactive, scalable and extensible microbiome data science using QIIME 2. *Nat. Biotechnol.* **37**, 852–857 (2019).

5. Edgar, R. C. Search and clustering orders of magnitude faster than BLAST. *Bioinformatics* **26**, 2460–2461 (2010).

6. Bolger, A. M., Lohse, M. & Usadel, B. Trimmomatic: a flexible trimmer for Illumina sequence data. *Bioinformatics* **30**, 2114–2120 (2014).

7. Andrews, S. FastQC. (2013).

8. Wood, D. E., Lu, J. & Langmead, B. Improved metagenomic analysis with Kraken 2. *Genome Biol.* **20**, 257 (2019).

9. Prjibelski, A., Antipov, D., Meleshko, D., Lapidus, A. & Korobeynikov, A. Using SPAdes De Novo Assembler. *Curr. Protoc. Bioinforma.* **70**, e102 (2020).

10. Seemann, T. Prokka: rapid prokaryotic genome annotation. *Bioinformatics* **30**, 2068–2069 (2014).

11. Altschul, S. F., Gish, W., Miller, W., Myers, E. W. & Lipman, D. J. Basic local alignment search tool. *J. Mol. Biol.* **215**, 403–410 (1990).

12. Page, A. J. *et al.* Roary: rapid large-scale prokaryote pan genome analysis. *Bioinformatics* **31**, 3691–3693 (2015).

13. Garber, A. I. *et al.* SprayNPray: user-friendly taxonomic profiling of genome and metagenome contigs. *BMC Genomics* **23**, 202 (2022).

14. Stamatakis, A. RAxML version 8: a tool for phylogenetic analysis and post-analysis of large phylogenies. *Bioinformatics* **30**, 1312–1313 (2014).

15. Guerrero-Araya, E., Muñoz, M., Rodríguez, C. & Paredes-Sabja, D. FastMLST: A Multi-core Tool for Multilocus Sequence Typing of Draft Genome Assemblies. *Bioinforma. Biol. Insights* **15**, 11779322211059238 (2021).

16. Deatherage, D. E. & Barrick, J. E. Identification of Mutations in Laboratory-Evolved Microbes from Next-Generation Sequencing Data Using breseq. in *Engineering and Analyzing Multicellular Systems: Methods and Protocols* (eds. Sun, L. & Shou, W.) 165–188 (Springer New York, 2014). doi:10.1007/978-1-4939-0554-6_12.

17. Oksanen, J. *et al.* *vegan: Community Ecology Package*. (2023).

18. Pagès, H., Aboyoun, P., Gentleman, R. & DebRoy, S. *Biostrings: Efficient manipulation of biological strings*. (2022).

19. Wickham, H., François, R., Henry, L., Müller, K. & Vaughan, D. *dplyr: A Grammar of Data Manipulation*. (2023).
